# Decoding Aging: Understanding the Complex Relationship among Aging, Free Radicals, and GSH

**DOI:** 10.1155/2020/3970860

**Published:** 2020-10-12

**Authors:** María E. López-Navarro, Mariana Jarquín-Martínez, Luis A. Sánchez-Labastida, Daniel Ramírez-Rosales, Marycarmen Godínez-Victoria, Laura Itzel Quintas-Granados, José Guadalupe Trujillo-Ferrara

**Affiliations:** ^1^Departamento de Bioquímica y Sección de Estudios de Posgrado e Investigación, Escuela Superior de Medicina del Instituto Politécnico Nacional, Plan de San Luis y Salvador Díaz Mirón s/n, Casco de Santo Tomás, Ciudad de México 11340, Mexico; ^2^Instituto Politécnico Nacional, ESFM, Av. Instituto Politécnico Nacional s/n, Edif. 9 U.P. Zacatenco, Col. San Pedro Zacatenco, Ciudad de México 07738, Mexico; ^3^Universidad Mexiquense de Bicentenario, Unidad de Estudios Superiores de Tultitlán, Av. Ex Hacienda de Portales s/n, Villa Esmeralda, Tultitlán, Estado de México 54910, Mexico

## Abstract

N-aryl maleimides can undergo a 1,4-Michael-type addition reaction with reduced glutathione (GSH), leading to a decreased concentration of GSH and an increased concentration of free radicals (FRs) in cells. GSH is a critical scavenging molecule responsible for protecting cells from oxidation and for maintaining redox homeostasis. N-aryl maleimides disturb redox homeostasis in cells because they scavenge thiol-containing molecules, especially GSH. This study aimed at measuring the concentrations of GSH and FRs by electronic paramagnetic resonance (EPR), in the brain and liver tissue of male Wistar rats (*ex vivo*) at different ages and after treatment with 3,5-dimaleimylbenzoic acid (3,5-DMB). Our results showed a relationship between age and the concentrations of GSH and FRs in cells. In young rats, the concentration of GSH was higher than in old rats, while the concentration of FRs was higher in adult rats than in young rats, suggesting an inverse relationship between GSH and FRs. On the other hand, the reaction of 3,5-DMB (an electrophilic maleimide) with cellular GSH increased the FR content. The results of this study contribute to the awareness that the process of aging implies not only a loss of tissue function but also essential changes in the molecular contents of cells, especially the concentrations of FRs and GSH.

## 1. Introduction

In the electron flow system, oxygen is the final single electron acceptor involved in the production of energy in the form of ATP. Cell respiration is balanced by the rates of free radical (FR) generation and elimination, which result from cell metabolism by single electron transfer. Some molecules lose or accept a single electron, leaving one or more unpaired electrons. Consequently, these molecules are highly reactive and unstable. The most abundant FRs in cells are reactive oxygen species (ROS) and reactive nitrogen species (RNS). Reactive oxygen and nitrogen species (RONS) refer to reactive radical and nonradical derivatives of oxygen and nitrogen, respectively. Tobacco and alcohol consumption, air and water contamination, certain drugs, industrial solvents, radiation, and a diet particularly rich in carbohydrates can also give rise to RONS in biological systems [1].

To reduce oxidized targets and thus diminish or avoid oxidative stress (OS), cells have potent antioxidant enzymes and nonenzymatic antioxidant systems. These enzymes include superoxide dismutase (SOD), peroxiredoxin, glutathione peroxidase (GPx), and catalase (CAT) [2], each with a distinct function. These enzymes may be sensors and transducers of oxidizing agents [3]. Other molecules participate in a nonenzymatic fashion in cells, such as ascorbic acid (vitamin C), *α*-tocopherol (vitamin E), glutathione (GSH), carotenoids, and flavonoids [2]. GSH is the major soluble antioxidant molecule in cells due to its abundance in the cytosol (1–11 mM), nucleus (3–15 mM), and mitochondrion (5–11 mM) of cells [2]. GSH, a reactive Cys residue containing tripeptide, is linked to glucose metabolism through the pentose phosphatase pathway. NADPH produced in this pathway maintains GSH in a reduced form [4].

The relationship between oxidants and the antioxidant system is the key to proper cell function, and the antioxidant system is triggered by signaling pathways in response to elevated levels of FRs and cell damage. OS occurs when oxidants and antioxidants are out of balance, provoking not only a disruption in redox signaling and control but also damage to molecules and cells [4, 5]. Hence, OS and FRs are linked to several age-related degenerative diseases, including cancer, stroke, and other cardiovascular and inflammatory diseases [6, 7]. According to the FR theory of aging, oxidants play an important role in the aging process. In childhood, the high level of thiols in cells creates a reducing environment that stimulates cell growth. In contrast, adult metabolism generates RONS, implying a considerable increase in the FR content in tissues.

A compound capable of selectively and covalently binding to thiols would inhibit increases in reducing environments, likely resulting in an increased level of FRs. In previous studies, our group found that GSH is scavenged by a series of aryl maleimides derived from benzoic acid, which are selective for thiol-containing compounds. Those aryl maleimides could be susceptible to nucleophilic attack at the carbonyl carbon and the olefinic carbons by a 1,2 addition reaction or a 1,4-Michael type addition reaction, respectively. Although both carbons are electrophiles, the highest value of local softness corresponds to olefinic carbons, indicating that they are more susceptible to a 1,4-Michael addition type reaction by thiol groups than are carbonyl carbons. This reactivity might be attributed to the soft base behavior of thiols [8]. The reaction of olefinic carbons with GSH leads to a significant decrease in the concentration of thiols in cells and the induction of apoptosis caused by a considerable increase in the level of FRs [8].

This study aimed at measuring the concentration of GSH and FRs in brain and liver tissues of male Wistar rats (*in vivo* and *ex vivo*) of different ages and after treatment with 3,5-dimaleimylbenzoic acid (3,5-DMB). The levels of FRs were determined with electronic paramagnetic resonance (EPR) which had been used to detect physiologic levels of specific species with a high specificity [9]. The reaction of 3,5-DMB (an electrophilic maleimide) with cellular GSH increased the FR content. A relationship was found between age and the concentration of GSH and FRs in cells. Young rats presented the highest levels of GSH and the lowest FRs levels. In contrast, old rats had the highest FR levels and the lower GSH amount, suggesting an inverse relationship between the GSH and FRs. The results of this study contribute to the awareness that the process of aging implies not only a loss of tissue function but also essential changes in the molecular contents of cells, especially the concentrations of FRs and GSH.

## 2. Materials and Methods

### 2.1. Synthesis of 3,5-Dimaleimylbenzoic Acid (3,5-DMB)

**Scheme 1:**
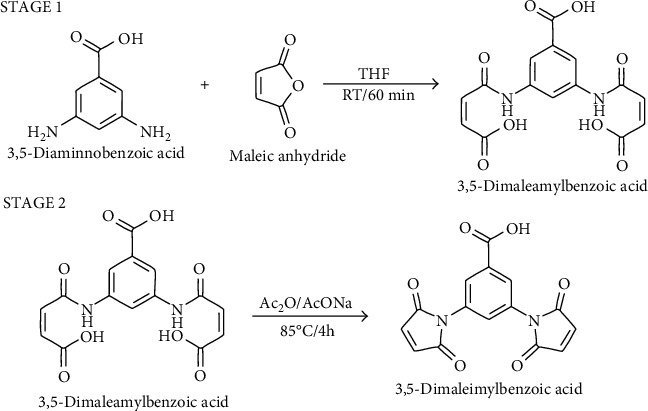
Synthesis of 3,5-dimaleimylbenzoic acid (3,5-DMB). Stage 1: 3,5-diaminobenzoic acid was reacted with maleic anhydride. Stage 2: cyclization of the precursor maleamide was achieved through dehydration with anhydrous sodium acetate to provide 3,5-DMB.

The synthesis of 3,5-DMB involved two steps (Scheme 1). First, the precursor 3,5-dimaleamylbenzoic acid was obtained by mixing 3,5-diaminobenzoic acid (25 mmol) with maleic anhydride (50 mmol) at a 1 : 2 ratio in anhydrous tetrahydrofuran (THF). The solution was incubated at room temperature under vigorous shaking for 1 h. The precipitate that formed was separated by filtration, washed with ethanol at 4°C, and dried at 40°C under vacuum.

Subsequently, 72 mmol of the precursor amide (maleamide) was added to 14 mmol anhydrous sodium acetate (AcONa) in 30 mL acetic anhydride (Ac_2_O). The mixture was incubated at 85°C with vigorous stirring for 4 h. The precipitate was removed by filtration, and the supernatant was maintained under gentle agitation at 4°C. Then, 100 mL of acidic water (pH 3) was added to induce precipitation. Finally, the precipitate that formed was filtered, washed with distilled water, and dried at 40°C before being characterized.

### 2.2. Characterization of 3,5-DMB

Compound purity was analyzed by thin-layer chromatography with a mixture of 1 : 1 ethanol : acetone. The product was characterized by 1H and 13C NMR as 3,5-DMB following a previous report [10]. Briefly, 1H- and 13C-NMR spectra were recorded on a Jeol GSX-270 (JEOL USA, Inc.) and Bruker Ascend g750 Ultrashield. First-order analysis of the 1H-NMR spin patterns was performed to obtain the chemical shifts (ppm) and coupling constants (Hz). 1H-NMR spectra were acquired at a spectral width of 5.9 kHz with 16 K data points based on an acquisition time of 2.73 s, a recycle delay of 2 s, a flip angle of 45° and 8 scans. ^13^C-NMR spectra were recorded with a spectral width of 25.1 kHz at 16 K data points, an acquisition time of 0.681 s, a flip angle of 45°, and 256 scans. 2D NMR spectra were captured on Jeol software at 295 K.

### 2.3. Characterization of the Reaction between 3,5-DMB and GSH

To determine whether a 1,4-Michael-type reaction occurred between 3,5-DMB and GSH, a solution of 3,5-DMB (1 mL of 0.1 M) in 0.01 M bicarbonate water (pH 8) was added to 1 mL of 0.2 M GSH or N-acetyl cysteine (NAC) at 5°C. Subsequently, thin layer chromatography was performed and revealed with 0.2% ninhydrin (2,2-dihydroxyindane-1,3-dione). The reaction was also monitored by VIS spectroscopy at 540 nm to distinguish among 3,5-DMB, GSH, and NAC. The reactions were characterized by ^1^H and ^13^C NMR at 270 MHz as mentioned above.

### 2.4. Global and Local Softness and Fukui Descriptors

Theoretical calculations were carried out as previously reported [8]. Briefly, the first potential ionization (*I*) and the electron affinity (*A*) were afforded by Gaussian 03 software and used to compute global parameters such as global hardness (*η*) (Equation (1)), chemical potential (*μ*) (Equation (2)), acceptor potential (*μ*^+^) (Equation (3)), donating potential (*μ*^−^) (Equation (4)), global softness (S) (Equation (5)), electrophilicity index (*ω*) (Equation (6)), electron-donating power (*ω*^−^) (Equation (7)), and electron-accepting power (*ω*^+^) (Equation (8)), according to the following equations:

(1) $$\begin{array}{c} \eta =\frac{I-A}{2}, \end{array}$$

(2) $$\begin{array}{c} \mu =-\frac{I+A}{2}, \end{array}$$

(3) $$\begin{array}{c} \mu^{+}=-\frac{I+3A}{4}, \end{array}$$

(4) $$\begin{array}{c} \mu^{-}=-\frac{3I+A}{4}, \end{array}$$

(5) $$\begin{array}{c} S=\frac{1}{\eta }, \end{array}$$

(6) $$\begin{array}{c} \omega =\frac{\mu^{2}}{2\eta }, \end{array}$$

(7) $$\begin{array}{c} \omega^{-}=\frac{{\left(\mu^{-}\right)}^{2}}{2\eta^{-}}, \end{array}$$

(8) $$\begin{array}{c} \omega^{+}=\frac{{\left(\mu \right)}^{2}}{2\eta^{+}}. \end{array}$$

The condensed Fukui functions were ascertained with Equation (9) and Equation (10):

(9) $$\begin{array}{c} \left({f_{x}}^{+}\right)=\left[q_{x}\;\left(N+1\right)-q_{x}\left(N\right)\right]\text{ for a reaction with a nucleophile} \end{array}$$

(10) $$\begin{array}{c} \left({f_{x}}^{-}\right)=\left[q_{x}\;\left(N\right)-q_{x}\left(N-1\right)\right]\text{ for a reaction with an electrophile} \end{array}$$

Where *q*_*x*_ represents the electronic population of *x* atom in the molecule.

Furthermore, local softness was determined by multiplying the value of the condensed Fukui function (*f*_*x*_^+/−^) by the global softness (*S*). For each atom in the *x* position of the molecule, local softness *s*_*x*_^+/−^ is expressed as Equation (11) and Equation (12):

(11) $$\begin{array}{c} s_{x}^{+}=\left({f_{x}}^{+}\right)\;S\text{ for a nucleophilic local attack} \end{array}$$

(12) $$\begin{array}{c} s_{x}^{-}=\left({f_{x}}^{-}\right)\;S\text{ for an electrophilic local attack} \end{array}$$

Theoretical calculations were performed for the electronic population by using Fukui descriptors, based on the quantum theory of atoms in molecules (QTAIM). The wave function for each of the neutral and ionic systems was computed using the optimized geometry for the neutral molecule. Finally, the electronic population was found using the AIM 2000 software, as previously described [8].

### 2.5. Biological Model and Experimental Design

Male Wistar rats served as the *in vivo* model. Rats were provided with a standard diet and water *ad libitum*. Animals were handled and maintained under the national guidelines on animal care, approved by the institutional Committee on Ethics in Research (registration # ESM-CICUAL-03/10-04-2019).

To study the 3,5-DMB effect on the GSH and FR, we selected to different organs, the brain, and the liver, because they have high mitochondrial amount, different metabolic requirements, both are FR producers and develop different complications and responses associated with redox homeostasis.

Control animals (*n* = 21) were monitored from 2-18 months of age. To examine the levels of GSH and FRs in liver and brain tissues, control subjects (*n* = 3) were analyzed at 2, 3, 5, 7, 8, 12, and 18 months of age.

Regarding the experimental group (*n* = 48), 3,5-DMB was intraperitoneally administered at 100 mg/kg body weight to 2- and 15-month-old animals, to determine the 3,5-DMB effect on young and old individuals in which the levels of GSH and FR are different. Tissue samples were collected at 1, 2, 4, 6, 8, 12, and 16 h posttreatment. Three control animals were analyzed before injection (time zero). For the determination of FRs, 0.15 g of brain tissue was obtained by frontal lobe dissection in the direction of the corpus callosum, hypothalamus, and hippocampus. GSH was evaluated with 1 g of damp brain tissue. The determination of the same two parameters was performed on equal amounts of damp liver tissue.

### 2.6. Quantification of GSH

GSH content was assessed *ex vivo* in liver and brain tissues from rats treated with 3,5-DMB and control (untreated) animals at several time points (each time and condition were assayed in triplicate). Tissues were extracted by grinding in the presence of 5% trichloroacetic acid (0.2 : 1.5 *w*(*g*)/*v*) and centrifugation at 1500 g for 30 min at room temperature. The supernatant was collected and immediately used to measure the level of GSH by the 5,5′-dithio-(2-nitrobenzoic acid) method [11] and read at 412 nm.

### 2.7. EPR Spectroscopic Analysis

Tissue samples were cut into 50 mg pieces and with an external diameter of 1 mm and were immediately frozen at -70°C and characterized at the same temperature on an X-band (9.3 GHz) EPR spectrometer (RADIOPAN, Poznan, Poland) with modulation of the magnetic field at 100 kHz. A single EPR spectrum was determined at 10^−4^ sec. To decrease the signal/noise level, spectra were accumulated 200-300 times. EPR spectra were recorded at the first derivative at room temperature and 72°C.

The amplitude (*A*), integral intensity (*I*), and linewidth (*ΔB*pp) values were determined from the EPR spectra using the ELF program of JAGMAR Firm (Kraków, Poland). The concentration of FRs in the samples is the value proportional to the integral of the intensity (*I*) of their EPR spectra. The *g*-factor was calculated from the resonance condition as follows (Equation (13)):

(13) $$\begin{array}{c} g=\frac{h\;v}{\mu B\;Br}, \end{array}$$

where *h* is the Planck constant, *v* is the microwave frequency, *μB* is the Bohr magneton and *Br* is the resonance magnetic field [12]. The *g*-factor is related to a stable FR content.

### 2.8. Statistical Analysis

Data were analyzed with SAS/STAT® software and are expressed as the mean ± SD. Data from EPR analysis and GSH quantification performed on tissue from three rats were used to calculate the mean and the standard deviation of FR and GSH levels, respectively. Normally distributed variables were examined with Student's *t*-test to determinate the possible correlation between FRs from the brain or liver and the age of rats.

## 3. Results

Synthesis of 3,5-DMB was achieved in 98% yield and 99% purity, in agreement with previously reported synthesis [8, 10, 13]. Structural analysis of the spectra indicated a close correspondence between the displacements and the composition/structure of 3,5-DMB.

Theoretical descriptors obtained from computational calculations of 3,5-DMB, NAC, cysteine, and GSH (Table 1) revealed that 3,5-DMB has the highest chemical potential (*μ*) and lowest global hardness (*η*). Accordingly, it was demonstrated in this study that 3,5-DMB selectively reacts with thiol groups from GSH.

**Figure 1:**
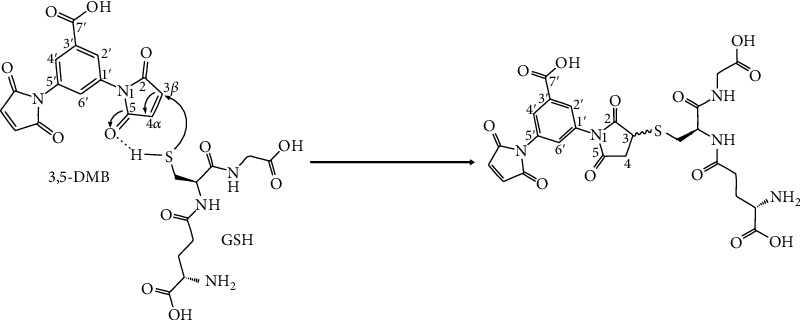
Proposed reaction mechanism for 3,5-dimaleimylbenzoic (3,5-DMB) acid with glutathione (GSH). The *α*,*β*-unsaturated carbonyl structure in 3,5-DMB acts as an electrophile, carrying out a 1,4-Michael-type reaction with the sulfur atom (S) from the thiol group of the cysteine residue in GSH (IUPAC-based numerical assignment). The *β*-carbon atom (C_3_) is shown as the preferred site of attack due to its positive polarization, whereas GSH acts as a Michael acceptor because of its high reactivity toward soft electrophiles, such as *α*,*β*-unsaturated compounds.

Additionally, an *in vitro* reaction was carried out between 3,5-DMB and NAC. According to thin layer chromatography (data not shown), COSY H-H (′H-H COrrelated SpectroscopY), COLOC (COrrelation through LOng-range Coupling) analysis, and ^1^H and ^13^C NMR at 270 MHz (Supplementary Figure S1), a 1,4-Michael type reaction takes place between 3,5-DMB and NAC, in which the *α*,*β*-unsaturated carbonyl structure in 3,5-DMB acts as an electrophilic compound (Figure 1). The reaction product is a 50-50% diastereoisomer mixture. Few changes occur in the imide moiety spectrum of 3,5-DMB due to reaction with NAC (compared to the spectrum reported for NAC alone). Moreover, aromatic moiety analysis of this reaction revealed a similar pattern as that found with the starting materials. However, there is a ^13^C signal at 39.54 ppm corresponding to C-S bond formation and a double of doubles signal at 4.19 ppm for each of the protons in the methylene moiety (Supplementary Figure S1) due to the diastereotopic nature of the reaction product.

### 3.1. Effect of Age on the Levels of GSH and Free Radicals (FRs) in Liver and Brain Tissue

According to the EPR spectra, the samples did not contain paramagnetic impurities. The spectra of all the analyzed samples revealed the presence of FRs (*g* = 2.003, Δ*H* = 1.0 mTl) (Supplementary Figure S2) that were stable (Table 2). The *g*-factor strongly indicates the presence of a high level of FRs.

**Figure 2:**
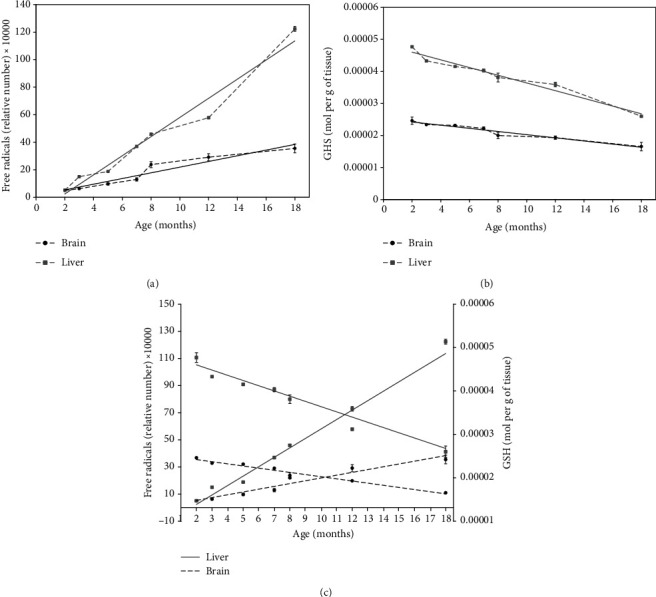
Effect of age on the levels of GSH and FRs. Male Wistar rats were used to obtain 1 g of brain (-•-) and liver (-∎-) tissue at 2-18 months of age (*n* = 3 for each age and tissue) to quantify the relative concentrations of FRs (a) and GSH (b). Circles and squares represent the mean ± standard deviation of three values from each sample extracted at a given measurement time. Experimental data were adjusted to a linear regression (solid lines). The statistical value (*r*^2^) is denoted in each polynomial regression plot. The solid lines indicate the experimental values for the liver (-∎-) tissue, and the dashed indicate the experimental values for the brain (-•-) tissue. Overall, the analysis indicates that with aging, the concentration of GSH decreases, and the FR content increases in both tissues (c).

An evaluation of the effect of age on the levels of GSH and FR in brain and liver tissues from rats is shown in Table 2 and Figure 2. Brain tissue displayed a 7-fold greater FR content in 18-month-old rats than that in 2-month-old rats (Table 2 and Figure 2(a)). Surprisingly, in liver tissue, the level of FRs was 23-fold higher in adult versus young rats (Table 2 and Figure 2(a)). In both liver and brain tissues, the concentration of FRs increased as a linear function of the age of rats. Experimental data were adjusted to a linear polynomial equation (*f*(*x*) = *ax* + *b*), where *a* is the slope and *b* is the intercept with the *y*-axis. Control experimental data fitted perfectly to a straight line (Figure 2) which allowed comparative analyzes. The *r*^2^ value was 0.9232 for brain tissue and 0.9639 for liver tissue (Figure 2(a)). The slope of the data was 3-fold greater for liver tissue (69525.28) than for brain tissue (20640.47). Statistical analysis demonstrated a strong correlation between FRs from the brain or liver and the age of rats (brain, *r*^2^ = 0.8244, *p* = 0.0001; liver, *r*^2^ = 0.9643, *p* = 0.0001). Thus, the level of FRs significantly increased in both tissues as rats aged and increased faster in the liver, probably due to the high metabolic rate in the liver and the importance of protecting the brain to allow several vital functions of the organism.

The concentration of GSH decreased in brain and liver tissues with age (Table 2 and Figure 2(b)). Young rats (2 months old) showed 1.5-fold and 1.8-fold higher levels of GSH in brain and liver tissues, respectively, than those in brain and liver tissues in adults (18 months old) (Figure 2(b)). Adjusting the experimental values to a linear equation afforded *r*^2^ values of 0.9522 for brain tissue and 0.9619 for liver tissue (Figure 2(b)). The slope of the data was 0.412-fold greater for liver tissue than for brain tissue, suggesting that the GSH content decreased faster in liver tissue.

To visualize the overall effect of age on the levels of GSH and FRs in brain and liver tissues, the adjusted experimental data were extrapolated from 0 to 36 months of age (Figure 2(c)), the average lifetime of a laboratory rat [14]. In very young individuals (<2 months old), the concentration of FRs was almost the same in brain and liver tissues, nearly zero (Figure 2(c)). Adult rats (>15 months old), on the other hand, had a 3.4-fold higher level of FRs in the liver than in the brain.

The concentration of GSH was 2-fold greater in liver tissue than in brain tissue in young animals (2 months old). With the aging of rats, a decrease in the GSH content was observed in both tissues and was 0.41-fold lower in liver tissue than in brain tissue (Figure 2(c)). Statistical analysis revealed a strong and negative correlation between the GSH content in both tissues and the age of rats (brain, *r*^2^ = 0.8817, *p* = 0.0001; liver, *r*^2^ = 0.9525, *p* = 0.0001). During the process of aging, the concentration of FRs increases, and that of GSH decreases in brain and liver tissues. The increase in FR content and the decrease in the concentration of GSH occur faster in liver tissue than in brain tissue (Figure 2(c)).

### 3.2. Treatment with 3,5-DMB Decreases the Level of GSH in Rats

**Figure 3:**
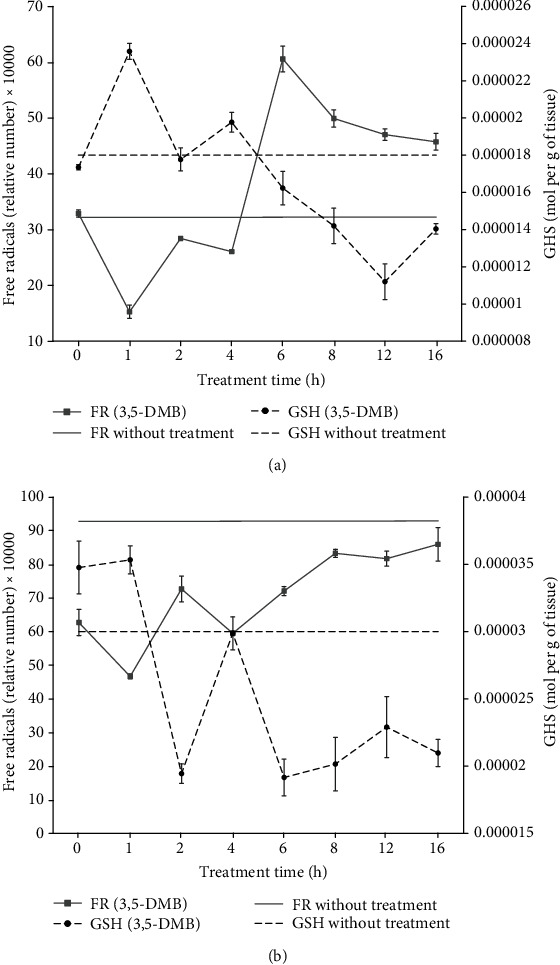
Effect of 3,5-DMB on the levels of GSH and FRs at 16 hours posttreatment in male Wistar rats. Evaluations were performed at 1, 2, 4, 6, 8, 12, and 16 h posttreatment with 3,5-DMB (*n* = 3). The relative number of FRs (-∎-) and concentration of GSH (-•-) were determined in 1 g of brain tissue (a) and liver tissue (b). The standard deviation is shown. Linear regression plots for FRs (gray solid line) or GSH (black dashed line) in untreated samples from brain or liver tissue (Figure 2) are included for comparison.

3,5-DMB treatment causes a loss of bright in the lens of treated rats but no weight loss was observed (data not shown). Brain tissue showed a significant increase (1.36-fold) in GSH content 1 h after treatment with 3,5-DMB, probably due to a specific cell reaction to maintain redox homeostasis. The level of GSH returned to the initial values by 2 h posttreatment and increases again (1.11-fold) at 4 h. From 4-12 h, the GSH content steadily decreased but increased again at 16 h (1.25-fold) (Figure 3(a)).

The concentration of FRs in brain tissue (Figure 3(a)) decreased by 2.15-fold at 1 h posttreatment, followed by an increase at 2 h, and a significant increase (2.33-fold) at 6 h. From 6-16 h, a steady decrease occurred, although these values were still higher than the basal level.

In liver tissue, a slight increase in GSH content was detected (1.02-fold) 1 h after treatment, followed by a significant decrease (1.82-fold) at 2 h and an increase (1.54-fold) at 4 h, reaching the basal value. There was another decrease (1.56-fold) at 6 h, an increase at 12 h (1.20-fold), and a decrease (1.09-fold) at 16 h (Figure 3(b)).

Regarding FRs, their concentration in liver tissue decreased (1.34-fold) at 1 h, increased (1.56-fold) at 2 h, decreased (1.22-fold) at 4 h, and increased again (1.44-fold) at 16 h (Figure 3(b)).

## 4. Discussion

Based on theoretical calculations, 3,5-DMB is an electrophile with a partial positive charge (soft acid), while GSH is a nucleophile with a local negative charge (soft base). The local softness (S_x_+/-) values of *α*,*β*-unsaturated compounds suggest that they are more susceptible to nucleophilic attack. The global and local reactivity indexes theoretically calculated from condensed Fukui functions (according to the published method) [8] point to the selectivity of 3,5-DMB for GSH. Since 3,5-DMB has greater chemical potential (*μ*) than GSH, the electron flow must go from the thiol group of GSH to the *α*,*β*-unsaturated carbonyl compound. Additionally, the fact that the lowest global hardness *η* = 3.0180 (Table 1) value was that of 3,5-DMB (versus that of GSH or NAC) reveals that it is most reactive as an electrophile among this group of compounds. According to its nucleophilicity, *ω* − = 6.0687 (Table 1), 3,5-DMB has a strong capacity to acquire electrons from the environment (until reaching saturation), considering the electrophilicity index (*ω*). Thus, the *α*,*β*-unsaturated carbonyl of 3,5-DMB appears to have electrophilic behavior (Table 1), and GSH (a thiol-containing compound) has nucleophilic behavior.

When an electron is transferred from GSH to 3,5-DMB, the latter acquires greater stability, as evidenced by the higher *ω* value for 3,5-DMB than that for GSH (Table 1). The electron-donating (*ω*−) and the electron-accepting (*ω*+) power indexes indicate the electron acceptor capacity of the *α*,*β*-unsaturated carbonyl (which has a small positive charge) and the electron donor capacity of GSH (which has a small negative charge). The present findings are in agreement with previous reports on the selectivity of aryl maleimides for thiol-containing compounds (unlike their reaction with other nucleophile groups) due to the selectivity of their vinyl moiety (soft acid) for thiol groups (soft base) [8]. Hence, the *α*-*β* unsaturated moiety is apparently the preferred site of nucleophilic attack because of its polarizability. Moreover, GSH is a highly reactive compound toward soft electrophiles (e.g., *α*,*β*-unsaturated compounds) and acts as a Michael-type acceptor [15, 16]. According to the theoretical data, olefinic carbonyl carbons selectively react with thiol-containing compounds. The reaction is highly regioselective but not stereoselective, generating two asymmetric chiral centers in a 50-50% diastereomeric mixture. Furthermore, theoretical calculations point to the selectivity of the reaction of thiol species with the C-3 position of the *α*,*β*-unsaturated group (but not the carbonyl group) of 3,5-DMB [8].

FRs are commonly quantified by indirect methods through the evaluation of metabolic pathways, such as fatty acid double bond oxidation. The current study directly measured FRs in tissue using EPR. Moreover, the *g*-factor value obtained from EPR studies gives important information about the magnetic properties of the FR species. A *g*-factor of approximately 2 serves as an indicator of FRs. In our study, the *g*-factor value was 2.003, that agrees with the value reported in the literature for C-O species [17]. Although the specific radical species in the samples were not identified, the spectra strongly suggested that the EPR signals correspond to FRs. Furthermore, considering that the *g*-factor values were from 2.003−2.004, the radicals were probably a carbon-oxygen bonded combination [17].

It should be possible to predict the perinatal FR content by adjusting experimental values from 0 to 36 months of age (the average lifetime of a laboratory rat) [14] to obtain a proper perspective regarding the inverse relationship between GSH and FRs in brain and liver tissue. Regarding the level of FRs as a function of aging, a sharp increase was found in the liver and only a moderate increase was found in the brain. The rat central nervous system is relatively immature at birth, but after 7 to 21 days of postnatal development, there is a substantial increase in the activity of metabolic pathways [18, 19], which may contribute to the increase in the FR content and the decrease in the concentration of GSH in the liver. The accumulation of dysfunctional aged mitochondria leading to the alteration of redox homeostasis cannot be discounted [20].

On the other hand, more endogenous protective mechanisms exist against FRs in the brain than in the liver, such as SOD (e.g., the catalytic activity of the isoforms Cu-Zn-SOD (SOD1) and Mn-SOD (SOD2)). These mechanisms are highly expressed in neurons [18] and possibly explain the lower increase in the level of FRs in the brain versus liver tissue. Since OS and cell damage occurs when the concentration of FRs exceeds the capacity of the scavenging system, the redox system is essential for the health of cells [18]. The only way to prevent cell damage or death is to strengthen the scavenging system, especially GSH. The current data reveal a rapid response of the antioxidant system to the imbalance between FR and GSH in brain tissue. This response likely involves a repair mechanism, physical defenses, and/or enzymatic antioxidant defenses (e.g., SOD, CAT, GPx, and GR) [2]. As observed with oxidation due to aging, treatment with 3,5-DMB (a thiol scavenger) produced a decrease in the level of GSH in a time-dependent manner after 3,5-DMB administration, resulting in a concomitant increase in the FR content of cells (Figure 3). Rapid response to a disturbance in redox homeostasis was anticipated for brain tissue because of its vital role in the organism. The significant decrease in the concentration of GSH in 15-month-old rats treated with 3,5-DMB led to a sharp and rapid increase in the FR content in both brain and liver tissues. Interestingly, a significant increase in the level of GSH was detected in brain tissue 1 h after 3,5-DMB treatment. At the same time, the level of FRs was lower in the brain tissue compared with liver tissue. Since 3,5-DMB decreased the level of thiols, the elevated GSH content at the early stages of treatment probably stemmed from an attempt by cells to maintain redox homeostasis. At 16 h posttreatment of adult rats, the concentration of GSH was 1.27-fold lower and the FR content was 1.42-fold higher than those in untreated samples, revealing that the imbalance in the adult rat brain was not completely repaired. According to a previous study, the immature brain of young rats is more vulnerable to OS in comparison to the mature adult brain [18]. The present finding of a 3,5-DMB-induced decrease in the level of GSH in young rats gives insight into how early-stage tissues face an elevated FR content, which is initially met with an increase of the concentration GSH (compared to that in untreated samples), similar to the response during the aging process. A daily dose of 3,5-DMB in young rats might cause that they age more quickly, generating “old juvenile-rats.” However, the 3,5-DMB effects on health, phenotype, and behavior need to be studied. On the other hand, it had been reported molecules that cause the opposite effect to 3,5-DMB treatment. One of these molecules is resveratrol that produces cell rejuvenation in the liver [21].

This initial 3,5-DMB-induced antioxidant response was more efficient in the brain than in the liver (Figure 3). Hence, the antioxidant response to a disturbance of redox homeostasis was more efficient in brain tissue than in liver tissue. It has been reported that GSH imbalance might cause diseases such as cancer, neurodegenerative diseases, cystic fibrosis, and HIV [22]. According to this, the systematic effect of 3,5-DMB on the organisms might cause a sustained GSH imbalance that may cause diseases.

It has been reported that there are approximately 1.1 x10^−9^ mol GSH/embryo [23] or 1.91 x10^−5^ mol GSH/g protein [24] in a Sprague-Dawley rat embryo (Wistar derived). Based on extrapolation from the current data, 3.02 x10^−4^ mol GSH/g brain tissue and 5.04 x10^−4^ mol GSH/g liver tissue should be found in a Wistar rat embryo, suggesting that FRs are tightly regulated by rapid changes in the GSH/GSSG relation.

## 5. Conclusions

Finally, we concluded that the thiol compound selectively reacts with the olefinic carbons of 3,5-DMB due to their local softness, and the reaction of thiol species with 3,5-DMB shows selectivity for vinylic carbon from the *α*,*β*-unsaturated group but not for the carbonyl carbon group. The reaction is not stereoselective, as evidenced by the clear multiplicity of signals in the spectra, indicating a diastereomeric mixture.

The current study attempts to provide insights into the relationship among FRs, reducing agents such as GSH, and the aging process. There is a relationship between age, the concentration of GSH, and the level of FRs. Young rats had a higher level of GSH and a lower level of FRs, while the opposite results were found in adult rats. Given the lower concentration of GSH and abrupt changes in the FR content in adult rats, GSH appears to be a regulable variable, and FRs appear to be a regulator variable, meaning that GSH might modulate the FR production. Despite thiols and FR have an important contribution to the systemic metabolism, there is an inverse relation between these two molecular families, thiols, and FR which is apparently a mechanism to recover redox homeostasis. However, the expression of the enzymes involved in redox homeostasis before and after 3,5-DMB administration and the nature of FR needs to be investigated.

## Figures and Tables

**Table 1:** Theoretical global and local descriptors for 3,5-DMB, N-acetyl cysteine, cysteine, and GSH.

Compound								
3,5-DMB	Global descriptors							
	*μ* (eV)	*μ*- (eV)	*μ* ^+^ (eV)	*η* (eV)	*S* (1/eV)	*ω* (eV)	*ω*- (eV)	*ω* ^+^ (eV)
	-5.1062	-6.8024	-3.4100	3.0180	0.1657	3.4198	6.0687	1.5254
	Local softness *s*_*x*_^+^							
	C=C (C3–C4)				0.0066			
	C=O				0.0046			

N-acetyl cysteine^1^	Global descriptors							
	*μ* (eV)	*μ*- (eV)	*μ* ^+^ (eV)	*η* (eV)	*S* (1/eV)	*ω* (eV)	*ω*- (eV)	*ω* ^+^ (eV)
	-3.8521	-6.2548	-1.4493	4.8055	0.1040	1.5439	4.0706	0.2186
	Local softness *s*_*x*_^−^							
	SH				0.0452			

Cysteine	Global descriptors							
	*μ* (eV)	*μ*- (eV)	*μ* ^+^ (eV)	*η* (eV)	*S* (1/eV)	*ω* (eV)	*ω*- (eV)	*ω* ^+^ (eV)
	-3.3075	-6.0074	-0.6076	5.3998	0.0926	1.0129	3.3417	0.0342
	Local softness *s*_*x*_^−^							
	SH				0.0318			

GSH	Global descriptors							
	*μ* (eV)	*μ*- (eV)	*μ* ^+^ (eV)	*η* (eV)	*S* (1/eV)	*ω* (eV)	*ω*- (eV)	*ω* ^+^ (eV)
	-3.6495	-5.8221	-1.4286	4.3936	0.1138	1.4957	3.8576	0.2322
	Local softness *s*_*x*_^−^							
	SH				0.0246			

3,5-DMB: 3,5-dimaleimylbenzoic acid, GSH: reduced Glutathione, *μ*: chemical potential, *μ*-: donating potential, *μ*^+^: acceptor potential, *η*: global hardness value, *S*: global softness, *ω*: Electrophilicity index, *ω*^−^: electron-donating power, *ω*^+^: electron-accepting power, eV: electronvolt. ^1^Global local indexes of reactivity for N-acetyl cysteine were taken from published data [8, 13].

**Table 2:** Quantification of free radicals and GSH in brain and liver tissue extracted from Wistar rats at various ages and treated with 3,5-DMB several times.

Age (months)	Tissue (1 g)	Relative number of FRs^1^	GSH content (mol/g)^2^
2	Brain	4.95 × 10^4^ ± 1.36 × 10^3^	2.46 × 10^−5^ ± 1.30 × 10^−7^
	Liver	5.10 × 10^4^ ± 0.88 × 10^3^	4.76 × 10^−5^ ± 1.12 × 10^−6^
3	Brain	6.24 × 10^4^ ± 0.94 × 10^3^	2.33 × 10^−5^ ± 1.86 × 10^−7^
	Liver	14.93 × 10^4^ ± 2.59 × 10^3^	4.32 × 10^−5^ ± 1.04 × 10^−7^
5	Brain	9.71 × 10^4^ ± 1.08 × 10^3^	2.31 × 10^−5^ ± 8.30 × 10^−8^
	Liver	18.75 × 10^4^ ± 4.10 × 10^3^	4.15 × 10^−5^ ± 2.51 × 10^−7^
7	Brain	12.92 × 10^4^ ± 2.78 × 10^3^	2.22 × 10^−5^ ± 4.81 × 10^−7^
	Liver	36.90 × 10^4^ ± 2.33 × 10^3^	4.03 × 10^−5^ ± 4.94 × 10^−7^
8	Brain	23.71 × 10^4^ ± 13.90 × 10^3^	1.99 × 10^−5^ ± 1.36 × 10^−6^
	Liver	45.87 × 10^4^ ± 2.82 × 10^3^	3.81 × 10^−5^ ± 1.00 × 10^−6^
12	Brain	29.10 × 10^4^ ± 4.37 × 10^3^	1.93 × 10^−5^ ± 7.14 × 10^−7^
	Liver	57.77 × 10^4^ ± 3.99 × 10^3^	3.59 × 10^−5^ ± 5.30 × 10^−7^
18	Brain	35.48 × 10^4^ ± 5.59 × 10^3^	1.65 × 10^−5^ ± 7.40 × 10^−8^
	Liver	122.34 × 10^4^ ± 14.75 × 10^3^	2.60 × 10^−5^ ± 1.33 × 10^−6^
Posttreatment time (h)			
0	Brain	32.88 × 10^4^ ± 6.51 × 10^3^	1.73 × 10^−5^ ± 1.36 × 10^−7^
	Liver	62.67 × 10^4^ ± 39.07 × 10^3^	3.47 × 10^−5^ ± 1.95 × 10^−6^
1	Brain	15.26 × 10^4^ ± 11.84 × 10^3^	2.35 × 10^−5^ ± 4.35 × 10^−7^
	Liver	46.68 × 10^4^ ± 7.44 × 10^3^	3.53 × 10^−5^ ± 1.03 × 10^−6^
2	Brain	28.42 × 10^4^ ± 0.85 × 10^3^	1.77 × 10^−5^ ± 6.17 × 10^−7^
	Liver	72.66 × 10^4^ ± 38.21 × 10^3^	1.94 × 10^−5^ ± 7.31 × 10^−7^
4	Brain	26.04 × 10^4^ ± 1.32 × 10^3^	1.97 × 10^−5^ ± 5.26 × 10^−7^
	Liver	59.50 × 10^4^ ± 6.71 × 10^3^	2.98 × 10^−5^ ± 1.23 × 10^−6^
6	Brain	60.57 × 10^4^ ± 23.13 × 10^3^	1.62 × 10^−5^ ± 8.95 × 10^−7^
	Liver	72.05 × 10^4^ ± 13.47 × 10^3^	1.91 × 10^−5^ ± 1.37 × 10^−6^
8	Brain	49.90 × 10^4^ ± 15.28 × 10^3^	1.42 × 10^−5^ ± 9.51 × 10^−7^
	Liver	83.25 × 10^4^ ± 12.16 × 10^3^	2.01 × 10^−5^ ± 1.98 × 10^−6^
12	Brain	47.00 × 10^4^ ± 10.20 × 10^3^	1.12 × 10^−5^ ± 9.59 × 10^−7^
	Liver	81.68 × 10^4^ ± 22.13 × 10^3^	2.29 × 10^−5^ ± 2.26 × 10^−6^
16	Brain	45.73 × 10^4^ ± 14.99 × 10^3^	1.40 × 10^−5^ ± 2.84 × 10^−7^
	Liver	85.94 × 10^4^ ± 49.69 × 10^3^	2.09 × 10^−5^ ± 1.01 × 10^−6^

^1^Data from EPR analysis performed on tissue from three rats (mean ± standard deviation). ^2^Data were calculated from tissue from three rats and are expressed as the mean ± standard deviation.

## Data Availability

Data used to support the findings of this study have been deposited in the Mendeley Data V1 repository (doi:10.17632/9n8r8m5kk7.1.

## Supplementary Materials

Supplementary Materials Herein, we investigate whether 3,5-DMB reacts with N-acetyl cysteine by NMR spectrometry, and according to our results, this reaction follows a 1,4-Michael type mechanism (Supplementary Materials). Furthermore, we measure the concentrations of reduced glutathione (GSH) and free radicals (FRs) in brain and liver tissue of male Wistar rats of different ages. The results showed that concentration of GSH was higher in young rats than that in old rats, while the concentration of FRs, measurement by electronic paramagnetic resonance (Supplementary Materials) was higher in adult rats than that in young rats, suggesting an inverse relationship between GSH and FRs. Similar relationships were found when thiol scavenger maleimide was used. Supplementary Figure S1: analysis of the reaction of 3′5′-DMB with N-acetyl cysteine. A fragment of the NMR spectrum for 3′5′-DMB is shown. For COSY H-H and 1H and 406 13C NMR at 270 MHz, the spectra show displacements that indicate a 1,4-Michael type mechanism. Ha and Hb correspond to hydrogen atoms Supplementary Figure S2: EPR spectra used to determine the number of free radicals. (A) Brain control sample, (B) liver control sample, and (C) brain sample of an animal treated with 3′5′-DMB.
