# Supplementary material for: Decoding Aging: Understanding the Complex Relationship among Aging, Free Radicals, and GSH
Source: Oxid Med Cell Longev. 2020 Oct 12;2020:3970860. doi: 10.1155/2020/3970860 (PMC7578726; doi:10.1155/2020/3970860)
Supplement: Supplementary Materials — Herein, we investigate whether 3,5-DMB reacts with N-acetyl cysteine by NMR spectrometry, and according to our results, this reaction follows a 1,4-Michael type mechanism (Supplementary Materials). Furthermore, we measure the concentrations of reduced glutathione (GSH) and free radicals (FRs) in brain and liver tissue of male Wistar rats of different ages. The results showed that concentration of GSH was higher in young rats than that in old rats, while the concentration of FRs, measurement by electronic paramagnetic resonance (Supplementary Materials) was higher in adult rats than that in young rats, suggesting an inverse relationship between GSH and FRs. Similar relationships were found when thiol scavenger maleimide was used. Supplementary Figure S1: analysis of the reaction of 3′5′-DMB with N-acetyl cysteine. A fragment of the NMR spectrum for 3′5′-DMB is shown. For COSY H-H and 1H and 406 13C NMR at 270 MHz, the spectra show displacements that indicate a 1,4-Michael type mechanism. Ha and Hb correspond to hydrogen atoms Supplementary Figure S2: EPR spectra used to determine the number of free radicals. (A) Brain control sample, (B) liver control sample, and (C) brain sample of an animal treated with 3′5′-DMB. [file 3970860.f1.pdf]

Supplementary Materials

Herein, we investigate whether 3,5-DMB reacts with N-acetyl cysteine by NMR spectrometry, and according to our results, this reaction follows a 1,4-Michael type mechanism (Supplementary Materials). Furthermore, we measure the concentrations of reduced glutathione (GSH) and free radicals (FRs) in brain and liver tissue of male Wistar rats of different ages. The results showed that concentration of GSH was higher in young rats than that in old rats, while the concentration of FRs, measurement by electronic paramagnetic resonance (Supplementary Materials) was higher in adult rats than that in young rats, suggesting an inverse relationship between GSH and FRs. Similar relationships were found when thiol scavenger maleimide was used.

Suplementary Materials

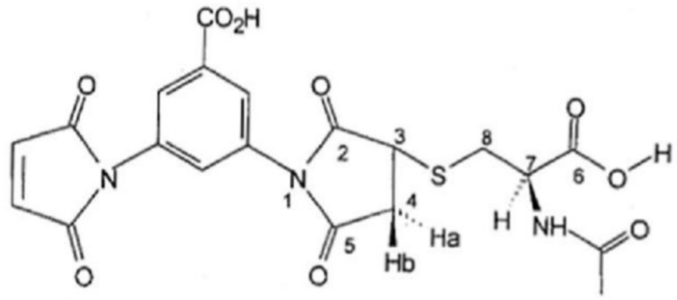

2

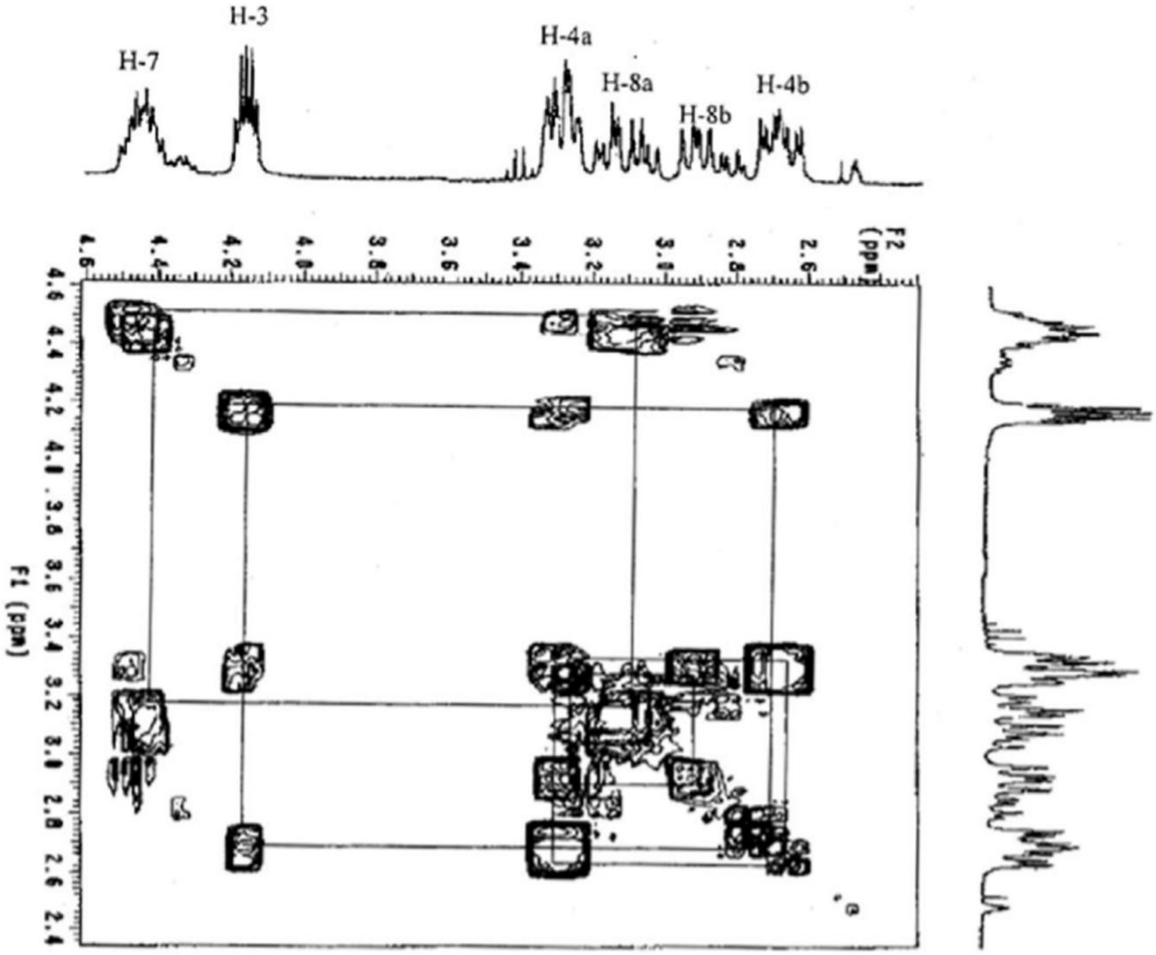

Supplemental figure S1. Analysis of the reaction of 3’5’-DMB with N-acetyl cysteine. A fragment of the NMR spectrum for 3’5’-DMB is shown. For COSY H-H and ^{1}H and ^{13}C NMR at 270 MHz, the spectra show displacements that indicate a 1,4-Michael type mechanism. Ha and Hb correspond to hydrogen atoms.

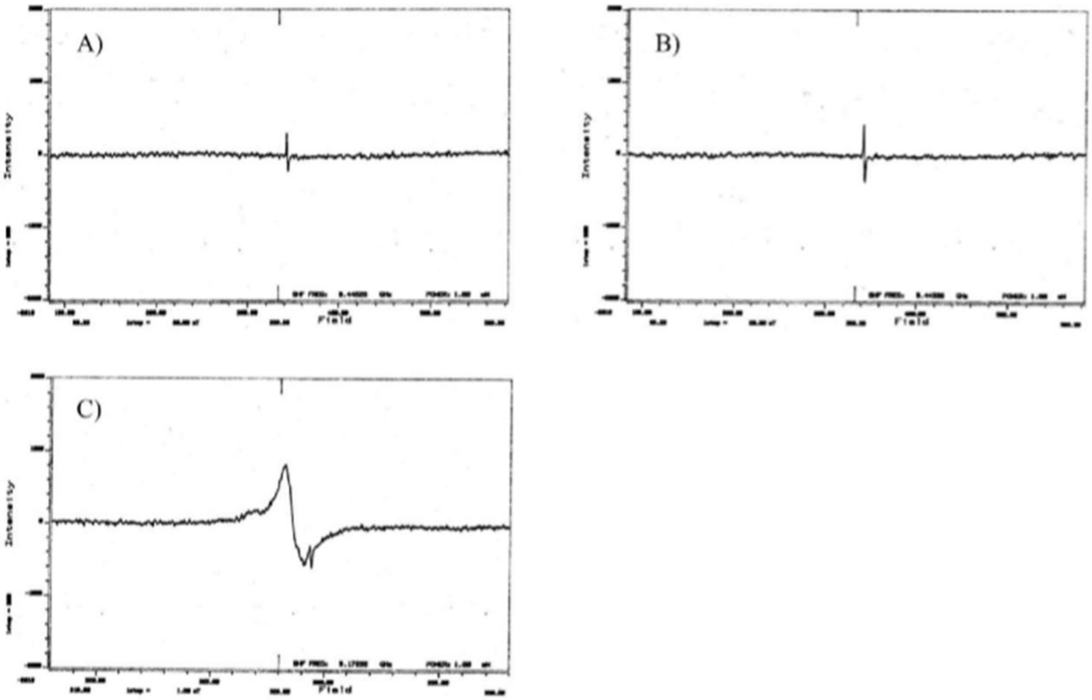

Supplemental figure S2. EPR spectra used to determine the number of free radicals. **A)** Brain control sample, **B)** liver control sample and **C)** brain sample of an animal treated with 3’5’-DMB.
